# Current evidence regarding the cellular mechanisms associated with cancer progression due to cardiovascular diseases

**DOI:** 10.1186/s12967-023-04803-2

**Published:** 2024-01-26

**Authors:** Tanawat Attachaipanich, Siriporn C. Chattipakorn, Nipon Chattipakorn

**Affiliations:** 1 Cardiac Electrophysiology Research and Training Center, Faculty of Medicine, Chiang Mai University, Chiang Mai, 50200 Thailand; 2 Center of Excellence in Cardiac Electrophysiology Research, Chiang Mai University, Chiang Mai, 50200 Thailand; 3 Neurophysiology Unit, Cardiac Electrophysiology Research and Training Center, Faculty of Medicine, Chiang Mai University, Chiang Mai, 50200 Thailand; 4 Department of Oral Biology and Diagnostic Sciences, Faculty of Dentistry, Chiang Mai University, Chiang Mai, 50200 Thailand; 5 Cardiac Electrophysiology Research Unit, Department of Physiology, Faculty of Medicine, Chiang Mai University, Chiang Mai, 50200 Thailand

**Keywords:** Reverse cardio-oncology, Myocardial infarction, Cardiac hypertrophy, Cancer, Micro-RNAs

## Abstract

Several large cohort studies in cardiovascular disease (CVD) patients have shown an increased incidence of cancer. Previous studies in a myocardial infarction (MI) mouse model reported increased colon, breast, and lung cancer growth. The potential mechanisms could be due to secreted cardiokines and micro-RNAs from pathological hearts and immune cell reprogramming. A study in a MI-induced heart failure (HF) mouse demonstrated an increase in cardiac expression of SerpinA3, resulting in an enhanced proliferation of colon cancer cells. In MI-induced HF mice with lung cancer, the attenuation of tumor sensitivity to ferroptosis via the secretion of miR-22-3p from cardiomyocytes was demonstrated. In MI mice with breast cancer, immune cell reprogramming toward the immunosuppressive state was shown. However, a study in mice with renal cancer reported no impact of MI on tumor growth. In addition to MI, cardiac hypertrophy was shown to promote the growth of breast and lung cancer. The cardiokine potentially involved, periostin, was increased in the cardiac tissue and serum of a cardiac hypertrophy model, and was reported to increase breast cancer cell proliferation. Since the concept that CVD could influence the initiation and progression of several types of cancer is quite new and challenging regarding future therapeutic and preventive strategies, further studies are needed to elucidate the potential underlying mechanisms which will enable more effective risk stratification and development of potential therapeutic interventions to prevent cancer in CVD patients.

## Introduction

Cardiovascular disease (CVD) is one of the leading causes of death worldwide. Over the past two decades, its prevalence has almost doubled from 271 to 523 million, and mortality rates continue to increase [1]. Cancer prevalence is also on the rise globally with an expected 28 million cases in 2040, nearly a 50% increase from 2020, and remains the leading cause of death [2]. Since the advancement of cancer treatment has led to more cancer survivors, there is increasing recognition of the devastating cardiovascular (CV) complications from cancer treatment. Cardio-oncology has emerged as a new field in an effort to mitigate the CV toxicity consequential to cancer therapy [3]. Interestingly, there is also accumulating evidence of a reverse relationship between CVD and cancer, termed “reverse cardio-oncology” [4]. Large cohort studies have demonstrated that patients with CVD have an increased risk of cancer development during follow-up [5–7]. In heart failure (HF) patients, non-CV death accounts for 15–30% of all deaths [8]. Cancer has been shown to be the leading cause of non-CV death in this population, contributing to approximately 40% [8].

Several cohort studies have demonstrated an increased risk of cancer in both HF and myocardial infarction (MI) patients [5–7, 9–13]. Additionally, a prospective cohort study in MI patients identified a subgroup that subsequently developed HF had an increased risk of cancer during follow-up [5]. Conversely, one retrospective study using the data from a Physicial Health Study (PHS) trial reported no association between HF and cancer among male physicians [14]. This discrepancy could be due the use of different study populations. These clinical reports are comprehensively summarized in Table 1.

**Table 1:** Clinical reports that demonstrated an association between cancer incidence and cardiovascular diseases

Model	Type of study/country/age/FU time	Number of CVD/number of cancer in CVD	Primary outcome	Interpretation	Refs.
HF	Retrospective cohort/Italy/mean 76/median 5.6 yr	103,421/12,036	HR 1.76 (95% CI 1.71–1.81) unadjusted	HF was associated with an increased risk of cancer	[10]
HF	Case–control/US/mean 73 yr/mean 7.7 yr	596/102	HR 1.60 (95% CI 1.14–2.26) adjusted for BMI, smoking, and Charlson comorbidity index	HF was associated with an increased risk of cancer	[6]
HF (LVEF < 45%)	Prospective cohort/Denmark/mean 67.8 yr/mean 4.5 yr	9307/975	IRR 1.24 (95% CI 1.15–1.33) adjusted for age and sex	HF was associated with an increased risk of cancer	[7]
HF	Retrospective cohort/Germany/mean 72.6 yr/0–10 yr	100,124/25,732	OR 1.76 (95% CI 1.71–1.81) unadjusted	HF was associated with an increased risk of cancer	[11]
HF post-MI	Prospective cohort/US/mean 72 yr/mean 4.9 yr	228/28	HR 2.16 (95% CI 1.39–3.35) adjusted for age, sex, and Charlson comorbidity index	Post MI patients who developed HF had increased risk of cancer	[5]
HF	Retrospective cohort/US/mean 61 yr/median 19.9 yr	1420/177	HR 1.02 (95% CI 0.84–1.25) adjusted for enrollment group, race, smoking, alcohol use, aspirin, family history of cancer, cirrhosis, PPI, H2 blocker, and sun exposure	HF was not associated with an increased risk of cancer among male physicians	[14]
MI	Prospective cohort/Norway/mean 62 yr/median 15.7 yr	1747/146	HR 1.46 (95% CI 1.21–1.77) adjusted for age, sex, BMI, SBP, DM, HDL, smoking, physical activity, and education level	MI was associated with an increased risk of cancer	[9]
MI	Retrospective cohort/Denmark/median male 59.2 yr, female 68.5 yr/0–17 yr	122,275/9769	IRR 1.08 (95% CI 1.03–1.13) adjusted for age, sex, calendar year, HT, DLP, DM, COPD, and socioeconomic status	MI was associated with an increased risk of cancer	[12]
MI	Retrospective cohort/Denmark/median male 63 yr, female 69 yr mean 5.9 yr	96,891/10,514	SIR 1.05 (95% CI 1.03–1.07) unadjusted	MI was associated with an increased risk of cancer	[13]

*COPD* chronic obstructive pulmonary disease, *DM* diabetes mellitus, *HF* heart failure, *HR* hazard ratio, *IRR* incidence rate ratio, *MI* myocardial infarction, *OR* odd ratio, *PHS* Physician Health study, *PPI* proton pump inhibitor, *SIR* Standardized incidence ratio, *yr* years

The nature of these clinical studies is evidently limited by their ability to establish a causal relationship between CVD and cancer, for example the increased incidence of cancer in CVD patients could be partly explained by shared risk factors, including obesity, diabetes mellitus, hypertension (HT), smoking, and inflammation [15]. However, emerging evidence particularly from in vivo studies suggests a plausible direct effect of CVD on the enhancement of tumor growth and metastasis. In this review, we aimed to comprehensively summarize the contemporary evidence on this reverse cardio-oncology concept and highlight the potential direct mechanism of CVD on the enhancement of tumor growth and metastasis from both in vitro and in vivo studies.

## Potential mechanisms of the effect of CVD on the enhancement of tumor proliferation and invasiveness: Evidence from in vitro and in vivo studies

### Effects of MI on tumor growth and metastases

In an in vivo study using APC^min^ mice, a genetically susceptible mouse strain prone to developing colonic adenomas, MI-induced HF in these mice led to left ventricular (LV) systolic dysfunction, hypertrophy and fibrosis. It was found that these mice had enhanced colon cancer growth [16]. To exclude the potential effects of hemodynamic disturbance, the study used a heterotopic heart transplant from an MI rat into other APC^min^ mice, which also resulted in increased colon cancer growth in the recipient mice [16]. Likewise, a study using MI mice with orthotopic breast cancer showed that MI enhanced breast cancer growth [17]. MI also enhanced breast cancer growth and metastasis in MMTV-PyMT mice, which was a transgenic mice model of spontaneous breast cancer [17]. In MI-induced HF mice with a xenograft Lewis lung carcinoma (LLC) model, it was shown that these mice also had enhanced lung cancer growth [18]. However, in a study using MI-induced HF mice with orthotopic renal cancer it was found that there was no effect on renal cancer growth and metastasis, despite the presence of LV systolic dysfunction, hypertrophy and fibrosis as in other studies [19]. These in vivo studies suggested there is a direct effect of MI on cancer growth, and the effects on tumors were potentially cancer-type specific.

Regarding the potential mechanisms, the effects of MI on tumor growth could be due to the cardiokines and mi-RNAs secreted from pathologic hearts [16, 18]. A study in an MI-induced HF model showed increased expression by cardiac mRNA of SerpinA3, SerpinA1, fibronectin (FN), ceruloplasmin (CP), and paraoxonase 1 (PON1) [16]. However, it was demonstrated that heterotopic transplantation of an MI heart resulted in only SerpinA3, FN and PON1 having increased expression in cardiac tissues [16]. To emphasize the importance of these cardiokines, an in vitro study demonstrated that incubation of colon cancer cells with SerpinA3 10 ng/mL or SerpinA1 50 ng/mL resulted in an enhanced proliferation of colon cancer cells. However, exposure to FN 20 mcg/mL, CP 0.1 mcM, or PON1 10 mM had no effect on colon cancer cell proliferation [16]. These findings indicated that the enhanced tumor growth in the MI-induced HF model could be due to secreted cardiokines, including SerpinA3 and SerpinA1. Unfortunately, the level of SerpinA3 in plasma and tumor tissues were not reported in that study [16].

A study in MI-induced HF mice with xenograft LLC demonstrated that the MI-induced HF condition mitigated the tumor sensitivity to ferroptotic cell death [18]. Ferroptosis is a regulated cell death pathway characterized by iron-dependent lipid peroxidation with a distinct morphological form of cell death [20, 21]. Growing evidence suggests the importance of ferroptosis in tumor biology in terms of its role in tumorigenesis, tumor progression, metastasis and therapeutic resistance, as a consequence of ferroptosis evasion [20, 21]. Additionally, ferroptosis is being recognized as a target of cancer vulnerability to cancer therapy, as it is a form of cell death observed in response to various cancer treatments [20, 21]. A study in xenograft LLC mice model showed that MI-induced HF in mice attenuated the effect of ferroptosis inducer, erastin and imidazole ketone erastin (IKE), on tumor growth [18]. The ferroptosis markers including prostaglandin-endoperoxide synthase 2 (PTGS2) and acyl-CoA synthase long-chain family member 4 (ACSL4) were upregulated, whereas glutathione peroxidase 4 (GPX4) was downregulated with erastin and IKE. These effects were attenuated in an MI-induced HF condition [18]. Interestingly, injection of isolated exosomes from MI mice also attenuated the effect of ferroptosis inducer on tumor growth in xenograft LLC mice [18]. An in vitro study exposed isolated exosomes from MI mice to a lung cancer cell line (LLC) and an osteosarcoma cell line (K7M2) demonstrated that those exosomes mitigated the erastin-induced ferroptosis in those cancer cells [18]. Moreover, the inhibitory effect of ferroptosis inducer on tumor cell invasion and migration was also attenuated by exosomes from MI mice [18]. In support of those findings on the roles of ferroptosis in tumor progression, Ferrostatin-1, a ferroptosis inhibitor, was shown to effectively reverse the inhibitory effect of erastin on tumor cell invasion and migration, which are further reversed by exosomes from MI mice [18].

The micro-RNAs (mi-RNAs) are short, non-coding RNA segments that regulate gene expression [22]. Further analysis revealed that in an MI-induced HF mouse with xenograft LLC model, the mice had increased levels of miR-22-3p in both the cardiac tissues and tumor and also in plasma [18]. However, precursor miR-22 (pre-miR-22) was only increased in cardiac tissues but not in tumor, indicating that miR-22-3p was released from the pathologic heart [18]. The potential effects of miR-22-3p were further evaluated in both in vitro and in vivo studies. An in vitro study using transfecting LLC cells with miR-22-3p showed cellular resistance to erastin-induced ferroptosis, whereas blocking the action of miR-22-3p further promoted ferroptosis in those cells [18]. Consistent with these findings, inhibition of cardiac-specific miR-22-3p in MI-induced HF mice with xenograft LLC treated with erastin effectively attenuated the effect of MI-induced HF on enhanced tumor growth [18]. These findings suggest that miR-22-3p secreted in the MI-induced HF model could play a role in promoting tumor growth by reducing the cellular sensitivity to ferroptosis, as well as potentially modulating tumor response to cancer therapy.

In addition to the potential effect of secreted cardiokines and mi-RNAs from a pathologic heart in enhancing tumor growth, immune cell reprogramming has also been proposed [17]. MI mice with an orthotopic breast cancer model showed an increase in Ly6C^hi^ monocytes in both plasma and tumor, as well as a decrease in T cells. However, the proportion of regulatory T cells in the tumor microenvironment was increased [17]. MMTV-PyMT mice with MI also had increased numbers of Ly6C^hi^ monocytes in the tumors [17]. In an adoptive transfer experiment, MI induced Ly6C^hi^ monocyte recruitment into the tumor was demonstrated. MI was induced in CCR2-diphtheria toxin receptor mice, which had depleted monocytes and showed decreased effects on tumor growth, decreased tumor Ly6C^hi^ monocytes, and a decrease in the proportion of regulatory T cells, as well as an increased proportion of activated T cells (granzyme B+) [17]. A bone marrow transplant from a donor MI mouse with tumor implantation into the wild type mice and implanted with a tumor also showed enhanced tumor growth and an increase in circulating Ly6C^hi^ monocytes [17]. These results indicated that the effect of MI on breast cancer growth and metastasis was through immune cell reprogramming and resulted in an immunosuppressive state within the tumor microenvironment [17].

**Fig. 1:**
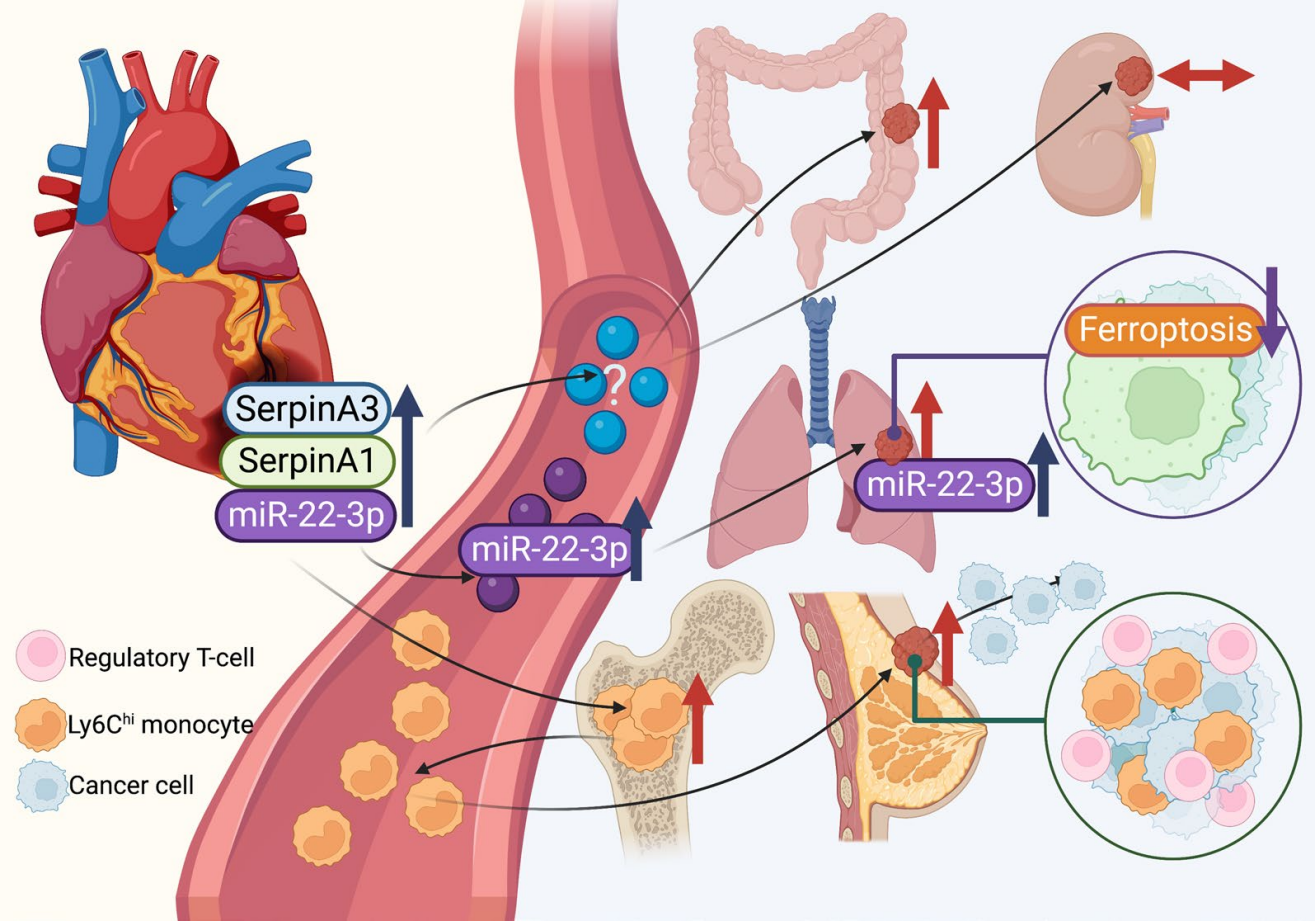
Potential direct effects of myocardial infarction (MI) on tumor growth and metastasis. An MI model was shown to promote colon, lung, and breast cancer growth. The MI condition also increased metastasis of breast cancer. Cardiac expression of SerpinA3, SerpinA1, and miR-22-3p were increased in MI models. The miR-22-3p was also increased in plasma and tumor tissue, resulting in attenuated breast cancer cell sensitivity to erastin-induced ferroptosis. MI also increased Ly6C^hi^ monocytes in plasma, tumor, and bone marrow, which led to an increased proportion of regulatory T-cells in the tumor microenvironment. Blue arrows indicate the changes of potential cardiokines and micro-RNAs. Red arrows indicate the effect of MI on tumor growth. The purple arrow indicates the change in ferroptotic cell death sensitivity. Figure created with BioRender.com

In conclusion, current evidence from in vitro and in vivo studies demonstrated that MI could enhance tumor growth and metastasis as a consequence of secreted cardiokines, and mi-RNAs and immune cell reprogramming. Interestingly, the effect of MI on tumor growth was shown to be cancer-type specific. These reports are comprehensively summarized in Fig. 1, Tables 2 and 3.

**Table 2:** Effect of myocardial infarction and cardiac hypertrophy on tumor progression: Evidence from in vivo studies

Model	Cardiac changes				Plasma biomarker changes	Tumor status				Interpretation	Refs.
	LV function	LVH	Fibrosis	mRNA expression	Protein and immune	Cancer type	Growth	Metastasis	mRNA expression and immune		
MI-induced HF APC^min^ mice	↓	↑	↑	↑SerpinA3, SerpinA1, FN, CP, PON1, CTGF	–	Colon	↑	–	–	HF enhanced colon cancer growth irrespective of hemodynamics through cardiac excreted factors	[16]
Heterotopic heart transplant of MI heart in APC^min^ mice into APC^min^ mice	↔	↑	↑	↑SerpinA3, FN, PON1 ↔ SerpinA1, CP	–	Colon	↑	–	–		
MI-induced HF mice with xenograft LLC	↓	–	–	↑miR-22-3p, pre-mi-R-22	↑miR-22-3p	Lung	↑↑	–	↑miR-22-3p ↔pre-mi-R-22	MI-induced HF enhanced tumor growth by attenuation of tumor sensitivity to ferroptosis via miR-22-3p	[18]
MI-induced HF mice with xenograft LLC + erastin 30 mg/kg/IP/OD	↓	–	–	–	–	Lung	↑	–	–		
MI-induced HF mice with xenograft LLC + IKE 30 mg/kg/IP/OD	↓	–	–	–	–	Lung	↑	–	–		
Xenograft LLC mice + erastin 30 mg/kg/IP/OD	↔	–	–	–	–	Lung	↓↓	–	–		
Xenograft LLC mice + erastin 30 mg/kg/IP/OD + Sham-EXO/Intra-tumor/q 48 h	↔	–	–	–	–	Lung	↓↓	–	–		
Xenograft LLC mice + erastin 30 mg/kg/IP/OD + Sham-EXO/Intra-tumor/q 48 h	↓	–	–	–	–	Lung	↓	–	–		
MI-induced HF in inhibited cardiomyocyte specific miR-22-3p mice with xenograft LLC + erastin 30 mg/kg/IP/OD	↓	–	–	↓ miR-22-3p	↓ miR-22-3p	Lung	↓	–	↓ miR-22-3p		
TAC-induced pressure overload mice with orthotopic breast cancer (PyMT)	↓	↑	↔	↑periostin, CTGF	↑periostin, CTGF ↔CD8^+^ T cell	Breast	↑	↔	↔CD8^+^ T cell	Early cardiac remodeling increased breast and lung cancer growth and metastasis possibly via periostin and CTGF	[23]
TAC-induced pressure overload mice + PyMT injection	↓	↑	↔	↑periostin, CTGF	↑periostin, CTGF	Breast	–	↑	–		
TAC-induced pressure overload mice with xenograft lung cancer (LLC)	↓	↑	↔	↑periostin, CTGF	↑periostin, CTGF ↔CD8^+^ T cell	Lung	↑	↔	↔CD8^+^ T cell		
TAC-induced pressure overload mice + LLC injection	↓	↑	↔	↑periostin, CTGF	↑periostin, CTGF	Lung	–	↑	–		
TAC-induced pressure overload NOD/SCID mice with orthotopic breast cancer	↓	↑	↔	↑periostin, CTGF	↑periostin, CTGF	Breast	↑	–	–		
TAC-induced pressure overload MCRR mice with orthotopic breast cancer	↔	↔	–	↔periostin, CTGF	↔periostin, CTGF	Breast	↔	–	–		
Low-dose PE induced hypertension mice with orthotopic breast cancer (PyMT)	↔	↑	↑	↑periostin, FN ↔CTGF, SerpinA3, SerpinE1, PON1, CP	↑periostin, FN, CTGF	Breast	↑	–	↑CTGF ↔periostin, FN, SerpinA3, SerpinE1, PON1, CP	Cardiac remodeling in the absence of contractile dysfunction was sufficient to promote breast cancer growth	[25]
ATF3-transgenic mice with orthotopic breast cancer (PyMT)	↓	↑	↑	↑periostin, SerpinA3, SerpinE1, CP, CTGF, FN	↔CP, CTGF, FN	Breast	↑	–	↑CTGF, FN ↔SerpinA3	Cardiac remodeling promoted tumor growth in ATF3-transgenic mice with breast and lung cancer models	[24]
ATF3-transgenic mice + PyMT injection	–	–	–	–	–	Breast	–	↑	–		
ATF3-transgenic mice with xenograft LLC	↓	↑	–	–	–	Lung	↑	–	–		
ATF3-transgenic mice with orthotopic breast cancer + doxycycline	–	↑	–	–	–	Breast	↑	–	–		
ATF3-transgenic mice + doxycycline + PyMT injection	–	–	–	–	–	Breast	–	↑	–		
MI-induced mice with orthotopic breast cancer (E0771)	↓	↑	–	–	↑Ly6C^hi^ monocyte	Breast	↑	–	↑Ly6C^hi^ monocyte ↓T cells ↑T_reg_ cells	MI enhanced tumor growth in breast cancer mice model via reprogramming of myeloid cells toward immunosuppressive state	[17]
CD45.2 mice implanted E0771 tumor + isolated Ly6C^hi^ monocytes from MI-induced CD45.1 non-tumor bearing mice	–	–	–	–	–	Breast	↔	–	↔Ly6C^hi^ CD45.1		
MI-induced CD45.2 mice implanted E0771 tumor + isolated Ly6C^hi^ monocytes from naive CD45.1 non-tumor bearing mice	–	–	–	–	–	Breast	↔	–	↑Ly6C^hi^ CD45.1		
MI-induced CCR2^DTR^ mice implanted E0771 tumor + DT injection (vs MI WT)	–	–	–	–	↓Ly6C^hi^ monocyte	Breast	↓	–	↓Ly6C^hi^ monocytes ↓T_reg_ cells ↑CD8^+^ T cell ↑CD8^+^GrB^+^		
Sham-operated CCR2^DTR^ mice implanted E0771 tumor + DT injection	–	–	–	–	↓Ly6C^hi^ monocyte	Breast	↔	–	↓Ly6C^hi^ monocytes ↔ T_reg_ cells ↑CD8^+^ T cell ↔ CD8^+^GrB^+^		
MI-induced mice implanted E0771 tumor + anti-CD8	–	–	–	–	–	Breast	↔	–	↓T cells		
Sham-operated mice implanted E0771 tumor + anti-CD8	–	–	–	–	–	Breast	↔	–	↓T cells		
CD45.1 mice + BM transplant from MI-induced CD45.2 mice implanted E0771 tumor + implanted E0771 after BM transplant (vs sham)	–	–	–	–	↑Ly6C^hi^ monocytes	Breast	↑	–	–		
MI-induced MMTV-PyMT mice	–	–	–	–	–	Breast	↑	↑	↑Ly6C^hi^ monocytes ↔ T cells ↔ T_reg_ cells		
MI-induced HF mice with orthotopic renal cancer (Renca cells)	↓	↑	↑	–	–	Renal	↔	↔	–	HF had neutral effect on renal cancer cell growth in MI-induced HF mice model	[19]

*ATF3* activating transcription factor 3, *BM* bone marrow, *CP* ceruloplasmin, *CTGF* connective tissue growth factor, *DT* diphtheria toxin, *EXO* exosomes, *FN* fibronectin, *GrB* granzyme B, *HF* heart failure, *IKE* imidazole ketone erastin, *IP* intraperitoneal, *LLC* Lewis lung carcinoma, *LVH* left ventricular hypertrophy, *MCRR* maladaptive cardiac remodeling-resistant, *MI* myocardial infarction, *MMTV* mouse mammary tumor virus, *NOD* nonobese diabetic, *PON1* paraoxonase 1, *PE* phenylephrine, *PyMT* Polyoma middle T, *OD* once daily, *SCID* severe combined immunodeficient, *TAC* transverse aortic constriction, *T*_*reg*_ regulatory T cells, *WT* wild-type

**Table 3:** Effect of myocardial infarction and cardiac hypertrophy on tumor progression: evidence from in vitro studies

Model	Exposure	Proliferation	Invasion/migration	Interpretation	Refs.
Colon cancer cells (HT-29)	SerpinA3/10 ng/mL	↑	–	SerpinA3 and SerpinA1 promoted colon cancer cell proliferation	[16]
	SerpinA1/50 ng/mL	↑	–		
	Fibronectin/20 mcg/mL	↔	–		
	Paraoxonase 1/10 mM	↔	–		
	Ceruloplasmin 0.1 mcM	↔	–		
Breast cancer cells (PyMT)	Serum of TAC-operated mice with PyMT model/48 h	↑↑	–	Periostin was increased in early cardiac remodeling in TAC mice and promoted breast and lung cancer cell proliferation	[23]
	Serum from TAC-operated mice without cancer/48 h	↑	–		
	Periostin 2000–4000 ng/mL/48 h	↑	–		
	Periostin 1000 ng/mL/48 h	↔	–		
	Periostin-depleted serum of TAC-operated mice with PyMT model/48 h	↔	–		
Lung cancer cells (LLC)	Serum from TAC-operated mice with LLC model/48 h	↑	–		
	Serum from TAC mice-operated mice without cancer/48 h	↑	–		
	Periostin 2000–4000 ng/mL/48 h	↑	–		
	Periostin 1000 ng/mL/48 h	↔	–		
PyMT cells	Serum from low-dose PE-infused mice/48 h	↑	–	Serum derived from PE-induced cardiac remodeling mice enhanced breast cancer cell proliferation	[25]
PyMT cells	Serum from AFT-3 transgenic mice/48 h	↑	–	Cardiac remodeling in AFT-3 transgenic mice without pressure overload increased breast and lung cancer cells growth and invasiveness	[24]
	Serum from AFT-3 transgenic mice + doxycycline/48 h	↑	–		
LLC cells	Serum from AFT-3 transgenic mice/48 h	↑	–		
LLC cells	Erastin/20 mcM/24 h	↓↓	↓↓	MI-derived EXO attenuated lung cancer and osteosarcoma cells	[18]
	Erastin/20 mcM/24 h + Sham-EXO 1 mcg/mL/24 h	↓↓	↓↓		
	Erastin/20 mcM/24 h + MI-EXO 1 mcg/mL/24 h	↓	↓		
Osteosarcoma cells (K7M2)	Erastin/5 mcM/24 h	↓↓	↓↓		
	Erastin/5 mcM/24 h + Sham-EXO 1 mcg/mL/24 h	↓↓	↓↓		
	Erastin/5 mcM/24 h + MI-EXO 1 mcg/mL/24 h	↓	↓		
LLC cells	Erastin/20 mcM/24 h	–	↓↓↓	MI-derived EXO further enhanced antiferroptotic activity of Fer-1 in erastin-induced suppression of invasion and migration	
	Erastin/20 mcM/24 h + Fer-1 2 mcM/24 h	–	↓↓		
	Erastin/20 mcM/24 h + Fer-1 2 mcM/24 h + Sham-EXO 1 mcg/mL/24 h	–	↓↓		
	Erastin/20 mcM/24 h + Fer-1 2 mcM/24 h + MI-EXO 1 mcg/mL/24 h	–	↓		
LLC cells	miR-22-3p mimics	↔	–	miR-22-3p attenuated erastin-induced ferroptosis	
	Erastin/20 mcM/24 h	↓↓	–		
	Erastin/20 mcM/24 h + miR-22-3p mimics	↓	–		
	AMO-22-3p	↔	–		
	Erastin/20 mcM/24 h	↓↓	–		
	Erastin/20 mcM/24 h + AMO-22-3p	↓↓↓	–		

*AMO* antisense oligonucleotide sequence, *ATF3* activating transcription factor 3, *EXO* exosomes, *Fer-1* ferrostatin-1, *LLC* Lewis lung carcinoma, *PE* phenylephrine, *PyMT* Polyoma middle T, *TAC* transverse aortic constriction

### Cardiac hypertrophy

A study using transverse aortic constriction (TAC) to induce pressure overload in mice resulted in LV hypertrophy and systolic dysfunction, leading to increased tumor growth in both orthotopic breast cancer (PyMT) and LLC models [23]. When breast and lung cancer cells were injected into TAC-operated mice, it was shown that cardiac remodeling also enhanced metastasis of both cancer types [23]. In a separate study using an immunodeficient mouse model lacking lymphoid cells and dysfunctional myeloid cells, TAC-operated NOD/SCID (nonobese diabetic/severe combined immunodeficiency) mice also demonstrated an increase in breast cancer growth [23]. However, there were no effects on tumor growth in the breast cancer model in TAC-operated maladaptive cardiac remodeling-resistant (MCRR) mice, which were resistant to cardiac remodeling and did not develop any cardiac remodeling [23].

Transgenic mice with overexpression of activating transcription factor 3 (ATF3) have been shown to develop cardiac remodeling, including LV hypertrophy and systolic dysfunction, and showed enhanced tumor growth in orthotopic breast cancer and xenograft lung cancer models [24]. Injection of breast cancer cells into ATF3-transgenic mice also increased breast cancer metastasis [24]. Inhibition of ATF3 expression caused by supplementation with doxycycline after cardiac remodeling had already occurred did not have any impact on tumor growth or metastasis, indicating that the enhanced tumor effect was due to cardiac remodeling and was independent of ATF3 expression [24]. Low-dose phenylephrine (PE)-induced HT in mice, which induced LV hypertrophy without systolic dysfunction, also enhanced orthotopic breast cancer growth [25]. These in vivo studies suggested that cardiac remodeling induced by TAC and genetic modification could potentially enhance tumor growth.

Several potential biomarkers have been found and reported on in models of cardiac hypertrophy in association with tumor progression. TAC-operated mice with orthotopic breast and lung cancer had shown enhanced tumor growth and it was found that cardiac expression and plasma levels of periostin and connective tissue growth factor (CTGF) were increased [23]. Similarly, in TAC-operated mice injected with breast cancer cells, increased cardiac and plasma levels of periostin and CTGF were observed, along with enhanced metastasis [23]. However, in TAC-operated MCRR mice with an orthotopic breast cancer model, which did not develop any cardiac remodeling and with no affect on tumor growth, there was no increase in cardiac or plasma levels of periostin and CTGF [23]. When serum from TAC-operated mice with or without breast and lung cancer was applied to breast cancer cells (PyMT) and lung cancer cells (LLC), increased cancer cell proliferation in both cell types was demonstrated [23]. In the same study, periostin at 2000 and 4000 ng/mL was shown to enhance the proliferation of both breast and lung cancer cells, whereas periostin-deprived serum from a TAC-operated mouse with a breast cancer model had no effect on cancer cell proliferation [23].

ATF3-transgenic mice with orthotopic breast cancer, which showed enhanced tumor growth, exhibited increased cardiac expression of periostin, CTGF, FN, SerpinA3, and CP [24]. The expression of CTGF and FN was also increased in breast cancer tissues, whereas the plasma levels of CTGF and FN remained unchanged [24]. When the serum from ATF3-transgenic mice was applied to breast and lung cancer cells, it was shown to enhance both breast and lung cancer cell proliferation [24]. In the low-dose PE-induced LV hypertrophy with orthotopic breast cancer model which resulted in increased the tumor growth, increased cardiac expression of periostin and FN was demonstrated [25]. The plasma levels of periostin, FN, and CTGF were also increased. However, in the tumor only the expression of CTGF was increased, while periostin and FN were unchanged. The SerpinA3, PON1, and CP expression also remained unchanged in both the cardiac tissues and tumor in this model. Consistently, serum from low-dose PE-infused mice was shown to enhance the proliferation of breast cancer cells [25].

**Fig. 2:**
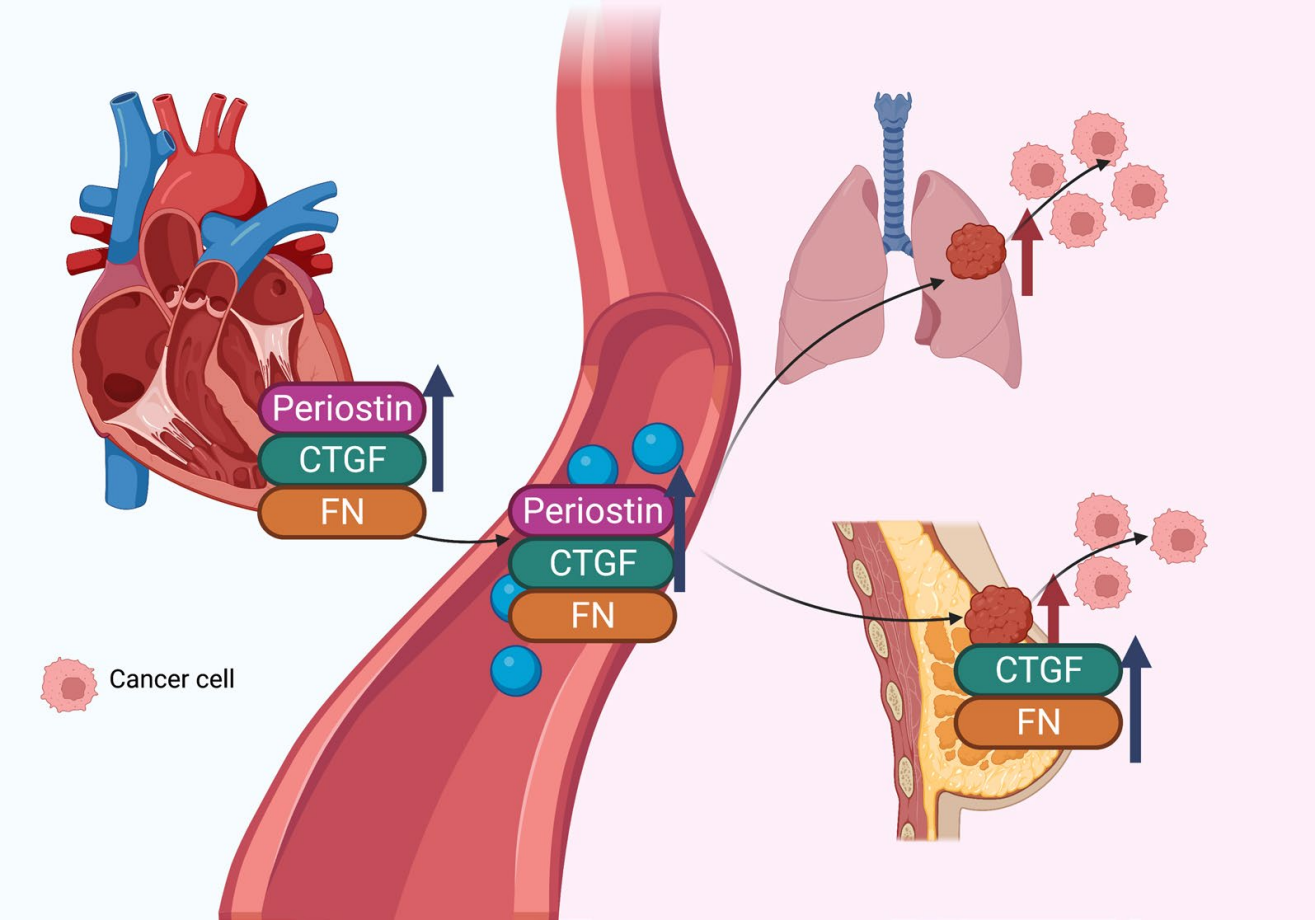
Potential direct effects of cardiac hypertrophy on tumor growth and metastasis. Cardiac hypertrophy was shown to enhance lung and breast cancer growth and metastasis. It has been reported that cardiac expression and plasma levels of periostin increase in a cardiac hypertrophy model. It has also been shown that expression of connective tissue growth factor (CTGF) and fibronectin (FN) increases in cardiac and tumor tissues and in plasma. Blue arrows indicate the changes of potential cardiokines. Red arrows indicate the effect of cardiac hypertrophy on tumor growth. Figure created with BioRender.com

All of these findings indicated that cardiac remodeling from various models could increase tumor growth and metastasis, which could be due to the secretion of factors including periostin, FN and CTGF. The reports regarding the potential mechanisms of the effect of CVD on cancer growth and metastasis from both in vivo and in vitro studies are comprehensively summarized in Fig. 2, and Tables 2 and 3.

## Potential cardiokines and mi-RNAs as potential links between CVD and cancer

Growing evidence suggests that several cardiokines and mi-RNAs could be responsible for the promotion of tumor proliferation and invasiveness in CVD models. These include SerpinA3, SerpinA1, periostin, miR-21, and miR-22. The effects and expression of those cardiokines and mi-RNAs in CVD and cancer based on in vitro, in vivo and clinical studies are comprehensively summarized in Tables 4 and 5.

**Table 4:** Potential cardiokines and microRNA for promotion of tumor progression in myocardial infarction and heart failure

Potential cardiokines and miR	Model	Cardiac expression	Plasma level	LV function	LVH	Outcome	Interpretation	Refs.
SerpinA3	*In vitro study*							
	HASMCs + ox-LDL 100mcg/mL/12 h	↑	–	–	–	–	Cardiac SerpinA3 expression was increased in aortic smooth muscle cells in atherosclerosis model	[27]
	*In vivo studies*							
	MI-induced HF APC^min^ mice	↑	–	↓	↑	–	Cardiac SerpinA3 expression was increased in MI-induced HF mice model	[16]
	Heterotopic heart transplant of MI heart in APC^min^ mice into APC^min^ mice	↑	–	↔	↑	–		
	ATF3-transgenic mice	↑	–	↓	↑	–	Cardiac SerpinA3 expression was increased in cardiac remodeling model using ATF3-transgenic mice	[24]
	Low-dose PE induced hypertension mice	↔	–	↔	↑	–	Cardiac SerpinA3 expression was not changed in cardiac remodeling without LV systolic dysfunction	[25]
	*Clinical studies*							
	CAD patients	–	↑	↔	–	–	Plasma SerpinA3 level was elevated in CAD patients and correlated with extension of coronary artery atherosclerosis	[27]
	MI patients	–	↑	↔	–	↑MACE	Plasma SerpinA3 level was elevated in MI patients, and was a predictor of MACE	[28]
	DCM patients	↑	↑	↓	–	↓survival	Plasma and cardiac SerpinA3 levels were elevated in DCM and associated with poor outcome in DCM patients	[29]
	HFrEF patients	–	↑	↓	–	↔survival	Plasma SerpinA3 level was elevated in HFrEF patients	[30]
	DCM patients pre LVAD implantation	↔	↑	↓	–	–	Plasma SerpinA3 level was elevated in HF patients and decreased after LVAD implantation	[31]
	DCM patients post LVAD implantation	↓	↔	–	–	–		
	Calcific AS patients	↑	↑	–	–	–	Plasma and cardiac SerpinA3 levels were elevated in calcific AS	[32]
SerpinA1	*In vivo study*							
	MI-induced HF APC^min^ mice	↑	–	↓	↑	–	Cardiac SerpinA1 expression was increased in MI-induced HF mice	[16]
	Heterotopic heart transplant of MI heart in APC^min^ mice into APC^min^ mice	↔	–	↔	↑	–		
	*Clinical studies*							
	MI patients	–	↑	–	–	↑survival	Plasma SerpinA1 level was elevated in MI patients, and was associated with improved prognosis	[39]
	HFrEF patients	–	↑	↓	–	↑NYHA	Plasma SerpinA1 level was increased in HFrEF patients, and was associated with higher NYHA class	[40]
Periostin	*In vitro study*							
	Adult rat cardiac fibroblasts + Ang II/10^–7^–10^–5^ M/24–48 h	↑	–	–	–	–	Ang II enhanced periostin expression in adult rat cardiac fibroblasts	[47]
	*In vivo studies*							
	MI mice	↑	–	–	–	–	Cardiac periostin expression was increased in MI mice	[49–51]
	Chronic Ang II-induced HT mice	↑	–	–	↑	–	Cardiac periostin expression was increased in chronic Ang II-induced LVH in mice	[47]
	High salt-induced HT rat	↑	–	–	–	–	Cardiac periostin expression was increased in high salt-induced HT rat model	[48]
	Aortic banding-induced HF mice	↑	–	↓	↑	–	Cardiac periostin expression was increased in hypertensive-induced cardiac remodeling	[52]
	*Clinical studies*							
	MI patients	↑	–	–	–	–	Cardiac periostin expression was increased in MI patients	[49]
	STEMI patients	–	↑ (vs lower group)	–	–	↓LVEF ↑CV events	Elevated plasma periostin level was associated with LVEF decline and increased CV events in STEMI patients	[54]
	HFrEF patients	↑	–	↓	–	–	Cardiac periostin expression was increased in HFrEF patients	[53]
	HFrEF patients on LVADs	↑ (vs off LVADs)	–	↓	–	–	Cardiac periostin expression was decreased after offload of LVADs in HFrEF patients	[52]
miR-21	*In vitro study*							
	Neonatal rat cardiomyocytes + PE/100 mcM	↑	–	–	–	–	miR-21 expression was increased in hypertrophic stimulated rat cardiomyocytes	[68, 70]
	Neonatal rat cardiomyocytes + LIF/1000 units/ml	↑	–	–	–	–		
	Neonatal rat cardiomyocytes + FBS/10%	↑	–	–	–	–		
	Neonatal rat cardiomyocytes + Ang II/1 mcM/48 h	↑	–	–	–	–	miR-21 expression was increased in Ang II-induced hypertrophy rat cardiomyocytes	[69]
	*In vivo studies*							
	Cardiac I/R mice	↑	–	–	–	–	Cardiac miR-21 expression was increased in a cardiac I/R mice model	[71]
	MI rat	↑ (border) ↓ (infarct)	–	–	–	–	Cardiac miR-21 expression was increased at border zone and decreased at infarct zone in MI rat model	[72]
	MI mice	↑	–	–	↑	–	Cardiac miR-21 expression was increased at infarct zone in MI mice model	[73]
	Thoracic aortic banding-induced cardiac hypertrophy mice	↑	–	–	↑	–	Cardiac miR-21expression was increased in cardiac hypertrophy mice	[68, 94]
	β_1_-adrenergic receptor transgenic mice with HF	↑	–	↓	–	–	Cardiac miR-21 expression was increased in HF mice	[70]
	TAC-induced HF mice	↑	–	↓	↑	–		
	Isoproterenol-induced HF mice	↑	–	↓	↑	–		
	*Clinical studies*							
	ACS patients	–	↑	–	–	–	Plasma miR-21 level was increased in ACS patients	[75]
	CAD patients	–	↑	–	–	–	Plasma miR-21 level was increased in CAD patients	[75]
	HFrEF patients	–	↑	↓	–	↓LVEF ↑NYHA	Plasma miR-21 level was increased in HFrEF patients and associated with decreased LVEF and increased NYHA	[74]
	HFrEF patients	↑	–	↓	–	–	Cardiac miR-21 expression was increased in HFrEF patients	[70]
miR-22	*In vitro studies*							
	Neonatal rat cardiomyocytes + PE + FBS	↑	–	–	–	–	miR-22 expression increased in PE-induced cardiomyocyte hypertrophy	[94]
	Neonatal rat cardiomyocytes + Ang II/1 mcM/48 h	↑	–	–	–	–	miR-22 expression was increased in Ang II-induced hypertrophy rat cardiomyocytes	[69]
	*In vivo studies*							
	MI mice	↑	↑	↓	–	–	Cardiac expression and plasma level of miR-22-3p were increased in MI nice model	[18]
	TAC-induced cardiac hypertrophy mice	↑	–	–	↑	–	Cardiac miR-22 expression was increased in early phase of TAC-induced cardiac hypertrophy mice	[94]
	*Clinical studies*							
	HFrEF patients	↑	–	↓	–	–	Cardiac miR-22 expression was increased in HFrEF patients	[95]
	HFrEF patients	–	↑	↓	–	↑CV death	Plasma miR-22 level was increased in HFrEF patients and associated with CV death	[96]
	HF patients	–	↑ (vs lower group)	–	–	↓CV events	Higher plasma miR-22-3p level was associated with lower frequency of CV events in HF patients	[97]
	CAD patients	–	↑	–	–	–	Plasma miR-22-3p level was increased in CAD patients	[98, 99]
	CAD patients	–	↓	–	–	–	Plasma miR-22 level was increased in CAD patients	[100]

*ACS* acute coronary syndrome, *Ang II* Angiotensin II, *AS* Aortic stenosis, *ATF3* activating transcription factor 3, *CAD* coronary artery disease, *CV* cardiovascular, *DCM* dilated cardiomyopathy, *FBS* fetal bovine serum, *HASMCs* Human aortic smooth muscle cells, *HFrEF* heart failure reduced ejection fraction, *HT* hypertension, *I/R* ischemic/reperfusion, *LIF* leukemia inhibitory factor, *LVADs* left ventricular assist device, *LV* left ventricular, *LVEF* left ventricular ejection fraction, *LVH* left ventricular hypertrophy, *MACE* Major adverse cardiac events, *MI* myocardial infarction, *miR* microRNA, *NMCMs* neonatal mouse cardiomyocytes, *NYHA* New York Heart Association, *ox-LDL* oxidized-LDL, *PE* phenylephrine, *STEMI* ST-elevation myocardial infarction

**Table 5:** Potential cardiokines and microRNA for the promotion of tumor progression

Potential cardiokines and miR	Model	Tumor expression	Plasma level	Growth	Metastasis/invasion/migration	Survival	Interpretation	Refs
Serpin A3	*In vitro studies*							
	Colon cancer cells (HT-29) + SerpinA3/10 ng/mL	–	–	↑	–	–	SerpinA3 enhanced colon cancer cell proliferation	[16]
	Colon cancer cells with high metastatic potential (HT-29LMM, KM-12L4)	↑ (vs low metastatic)	–	–	–	–	SerpinA3 expression was higher in colon cancer cells with higher metastatic potential and associated with colon cancer cell migration and invasion	[34]
	HT-29LMM, KM-12L4 + downregulated SerpinA3	↓	–	–	↓	–		
	Breast cancer cells (MDA-MB-231, BT549, MCF-7, T-47D) + upregulated SerpinA3	↑	–	↑	↑	–	SerpinA3 enhanced breast cancer cell invasion and migration	[35]
	MDA-MB-436 + downregulated SerpinA3	↓	–	↓	↓	–		
	Lung adenocarcinoma cells (CADO-LC11, LC29, LC45)	↑	–	–	–	–	SerpinA3 expression was increased in lung adenocarcinoma cells	[33]
	Lung non-adenocarcinoma cells (OC-35, OC-10, CADO-LC22, CADO-LC3, CADO-LC15)	↔	–	–	–	–		
	GBM cells (U251MG) + downregulated SerpinA3	↓	–	–	↓	–	SerpinA3 enhanced GBM cell invasion	[36]
	*In vivo studies*							
	MI-induced HF APC^min^ mice	–	–	↑	–	–	Increased SerpinA3 cardiac expression in MI-induced HF was associated with increased tumor growth in APC^min^ mice model	[16]
	Heterotopic heart transplant of MI heart in APC^min^ mice into APC^min^ mice	–	–	↑	–	–		
	ATF3-transgenic mice with PyMT	↔	–	↑	–	–	Cardiac remodeling in ATF3-transgenic mice did not increase SerpinA3 expression, but enhanced breast cancer growth	[24]
	Low-dose PE induced hypertension mice with PyMT	↔	–	↑	–	–	Cardiac remodeling without LV systolic dysfunction did not increase Serpin A3 expression, but enhanced breast cancer growth	[25]
	Mice + downregulated SerpinA3 colon cancer cells (HT-29LMM)	↓	–	–	↓	–	SerpinA3 regulated liver metastasis of colon cancer in a mouse model	[34]
	*Clinical studies*							
	Colon cancer patients	↑ (vs negative)	–	↔	↑	–	Increased SerpinA3 expression in colon cancer tissue was associated with metastasis	[34]
	Lung cancer patients	↑ (vs negative)	–	↑	↔	↓	Increased SerpinA3 expression in lung cancer tissue was associated with larger tumor size and poor survival	[33]
	Lung cancer patients	–	↑	–	↑	–	Plasma SerpinA3 levels was increased in lung cancer patients and associated with metastasis	[38]
	Breast cancer patients	↑	–	–	–	–	SerpinA3 expression was increased in breast cancer tissue	[35]
	Glioma patients	↑	–	–	–	↓	SerpinA3 expression was increased in brain glioma tissue and associated with poor survival	[36]
Serpin A1	*In vitro studies*							
	Colon cancer cells (HT-29) + SerpinA1/50 ng/mL	–	–	↑	–	–	SerpinA1 enhanced colon cancer cell proliferation	[16]
	Gastric cancer cells (AGS, MKN45) + upregulated SerpinA1	↑	–	–	↑	–	SerpinA1 promoted gastric cancer cell migration and invasion	[44]
	AGS, MKN45 + downregulated SerpinA1	↓	–	–	↓	–		
	Colon cancer cells (DLD-1, SW-480) + upregulated SerpinA1	↑	–	–	↑	–	SerpinA1 promoted colon, breast and ovarian cancer cell invasion and migration	[42]
	DLD-1, SW-480 + downregulated SerpinA1	↓	–	–	↓	–		
	Breast cancer cell (MCF-7, MDA-MB-231) + upregulated SerpinA1	↑	–	–	↑	–		
	MCF-7, MDA-MB-231 + downregulated SerpinA1	↓	–	–	↓	–		
	Ovarian cancer cells (A2780, SKVO3) + upregulated SerpinA1	↑	–	–	↑	–		
	A2780, SKVO3 + downregulated SerpinA1	↓	–	–	↓	–		
	Lung adenocarcinoma cell with high metastatic potential (CL1-5)	↑ (vs low metastatic CL1-0)	–	–	–	–	SerpinA1 expression was higher in lung adenocarcinoma cells with higher metastatic potential and associated with cell migration and invasion	[43]
	CL1-5 + downregulated SerpinA1	↓	–	–	↓	–		
	CL1-0 + upregulated SerpinA1	↑	–	–	↑	–		
	Lung adenocarcinoma cells (A549, SPC-A1) + upregulated SerpinA1	↑	–	–	↑	–	SerpinA1 promoted lung cancer cell migration	[45]
	A549, SPC-A1 + downregulated SerpinA1	↓	–	–	↓	–		
	NSCLC cells (H661) + upregulated SerpinA1	↑	–	↑	↑	–	SerpinA1 promoted NSCLC cell proliferation and migration	[41]
	NSCLC cells (H1975) + downregulated SerpinA1	↓	–	↓	↓	–		
	*In vivo study*							
	Mice + lung adenocarcinoma cells (CL1-5)	↑	–	–	↑	–	SerpinA1 promoted lung metastasis in lung adenocarcinoma mice	[43]
	Mice + downregulated SerpinA1 CL1-5	↓	–	–	↔	–		
	*Clinical studies*							
	Gastric cancer patients	↑ (vs negative)	–	↑	↑	↓	SerpinA1 expression was associated with tumor size, lymph node metastasis and poor survival in gastric cancer patients	[44]
	Colorectal cancer patients	↑ (vs negative)	–	↑	↑	↓	SerpinA1 expression was associated with tumor size, metastasis and poor survival in colorectal cancer patients	[42]
	Lung adenocarcinoma patients	↑(vs negative)	–	↔	↑	↓	SerpinA1 expression was associated with lymph node metastasis and poor survival in lung adenocarcinoma patients	[43, 45]
	NSCLC patients	↓	–	–	–	↓	SerpinA1 expression was decreased in NSCLC tumor tissue and was associated with poor survival	[41]
Periostin	*In vitro studies*							
	Breast cancer cells (PyMT) + Periostin 2000–4000 ng/mL/48 h	–	–	↑	–	–	Periostin enhanced breast and lung cancer cell proliferation	[23]
	PyMT + Periostin 1000 ng/mL/48 h	–	–	↔	–	–		
	Lung cancer cells (LLC) + Periostin 2000–4000 ng/mL/48 h	–	–	↑	–	–		
	LLC + Periostin 1000 ng/mL/48 h	–	–	↔	–	–		
	Colon cancer cells (CX-1NS) in serum depleted condition + upregulated periostin	↑	–	–	–	–	Periostin promoted colon cancer cell survival in conditions of stress	[58]
	Breast cancer cells (MCF-7) + upregulated periostin	↑	–	–	–	–	Periostin promoted angiogenesis in breast cancer cells	[57]
	NSCLC cells (A549) + upregulated periostin	↑	–	↑	↑	–	Periostin enhanced NSCLC cell proliferation and migration	[56]
	*In vivo studies*							
	Mice + upregulated periostin colon cancer cells (CX-1NS)	↑	–	–	↑	–	Periostin enhanced colon cancer growth and metastasis in mouse model	[58]
	SCID mice + upregulated periostin breast cancer cells (MDA-MB-231)	↑	–	↑	–	–	Periostin enhanced breast cancer growth in an SCID mouse model	[57]
	*Clinical studies*							
	Colon cancer patients	↑	↑	–	↑	↓	Plasma periostin and tumor expression were increased in colon cancer and associated with metastasis and poor survival	[58, 59]
	Breast cancer patients	↑	–	↔	↔	↓	Tumor periostin expression was increased in breast cancer and associated with poor survival	[57, 60]
	Breast cancer patients	–	↑	↔	↑	–	Plasma periostin level was higher in breast cancer with bone metastasis	[66]
	NSCLC patients	↑	↑	↔	↔	↓	Plasma periostin level and tumor expression were elevated in NSCLC patients and associated with poor survival	[56, 64]
	HCC patients	↑ (vs low level)	–	↔	↑	↓	Higher periostin expression in HCC was associated with metastasis and poor survival	[62]
	HCC patients	–	↑	↔	↔	↓	Plasma periostin level was increased in HCC patients and associated with poor survival	[65]
	Prostate cancer patients	↑	–	–	↑	↓	Tumor periostin expression was increased in prostate cancer and associated with advanced stages and poor survival	[61, 63]
miR-21	*In vitro studies*							
	Colorectal cancer cells (HCT-116, SW480) + upregulated miR-21	↑	–	↑	↑	–	miR-21 promoted colorectal cancer cell proliferation and invasion	[78]
	HCT-116, SW480 + downregulated miR-21	↓	–	↓	↓	–		
	Colorectal cancer cells (RKO) + downregulated miR-21	↓	–	–	↓	–	miR-21 promoted colorectal cancer cell invasion and metastasis	[81]
	Breast cancer cells (BCAP-37, MCF-7, MDA-MB-231, MDA-MB-435)	↑	–	–	–	–	miR-21 increased expression in breast cancer cells and associated with cancer invasiveness	[82]
	MDA-231 + upregulated miR-21	↑	–	–	↑	–		
	MDA-231 + downregulated miR-21	↓	–	–	↓	–		
	MDA-435 + downregulated miR-21	↓	–	–	↓	–		
	MCF-7 + upregulated miR-21	↑	–	–	↑	–	miR-21 promoted breast cancer cell invasion and migration	[83]
	NSCLC cells (H2170, A549, SPC-A1)	↑	–	–	–	–	miR-21 expression was increased in NSCLC cells and associated with proliferation, migration and invasion	[79]
	A549, H2170 + upregulated miR-21	↑	–	↑	↑	–		
	A549, H2170 + downregulated miR-21	↓	–	↓	↓	–		
	Gastric cancer cells (BGC-823) + upregulated miR-21	↑	–	↑	↑	–	miR-21 promoted gastric cancer cell growth and invasion	[80]
	BGC-823 + downregulated miR-21	↓	–	↓	↓	–		
	Glioblastoma cells (A172, U87, U373, LN229, LN428, LN308)	↑	–	–	–	–	miR-21 expression was increased in glioblastoma cell lines	[84]
	Glioblastoma cells (A172, U87) + downregulated miR-21	↓	–	–	↓	–	miR-21 enhanced glioblastoma cell invasion	[85]
	HCC cells (HepG2, SK-HEP-1, SNU182, SNU449, PLC/PRF-5)	↑	–	–	↑	–	miR-21 expression was increased in HCC cells and associated with invasiveness	[86]
	HepG2, SK-HEP-1, SNU182, PLC/PRF-5 + downregulated miR-21	↓	–	–	↓	–		
	DLBCL cells (CRL-2630)	↑	–	–	–	–	miR-21 expression was increased in DLBCL cells	[87]
	*In vivo studies*							
	Mice + upregulated miR-21 colon cancer cells (HCT-116)	↑	–	↑	–	–	miR-21 promoted colorectal cancer growth in mouse model	[78]
	Mice + downregulated miR-21 HCT-116	↓	–	↓	–	–		
	Mice + downregulated miR-21 breast cancer cells (MCF-7)	↓	–	↓	–	–	miR-21 promoted breast cancer growth in mouse model	[83]
	*Clinical studies*							
	Colorectal cancer patients	↑	–	–	↑	–	miR-21 expression was increased in colorectal cancer and associated with metastasis	[78]
	Colon cancer patients	↑	–	–	–	↓	miR-21 expression was increased in colon cancer and associated with poor survival in colon cancer patients	[89]
	Breast cancer patients	↑	–	–	↑	–	miR-21 expression was increased in breast cancer and associated with metastasis	[82, 88]
	Breast cancer patients	–	↑	–	–	–	Plasma miR-21 levels were increased in breast cancer patients	[83]
	NSCLC patients	–	↑	–	↑	↓	Plasma miR-21 level was increased in NSCLC patients and associated with lymph node metastasis and poor survival	[90]
	NSCLC patients	↑	–	–	↑	–	Tumor miR-21 expression was increased in NSCLC and associated with lymph node metastasis	[79]
	Lung cancer patients	↑	–	–	–	–	Tumor miR-21 expression was increased in lung cancer tissue	[88]
	Prostate cancer patients	↑	–	–	–	–	Tumor miR-21 expression was increased in prostate cancer tissue	[88]
	Prostate cancer patients	–	↑	–	↑	–	Plasma miR-21 level was increased in prostate cancer patients and associated with metastasis	[92]
	Gastric cancer patients	–	↑	–	–	–	Plasma miR-21 level was increased in gastric cancer patients	[91]
	Gastric cancer patients	↑	–	–	↑	–	Tumor miR-21 expression was increased in gastric cancer and associated with lymph node metastasis	[80]
	Glioma patients	↑	–	–	–	–	Tumor miR-21 expression was increased in glioma	[85]
	HCC patients	↑	–	–	–	–	Tumor miR-21 expression was increased in HCC	[86]
	DLBCL patients	↑	–	–	–	–	Tumor miR-21 expression was increased in DLBCL	[87]
miR-22	*In vitro studies*							
	NSCLC cells (A549, H1299)	↓	–	–	–	–	miR-22 expression was decreased in NSCLC cell lines and suppressed cancer cell proliferation and migration	[102]
	A549, H1299 + Overexpressed miR-22	↑	–	↓	↓	–		
	NSCLC cells (H1975, H1299) + transfected miR-22-3p	↑	–	↓	–	–	miR-22-3p inhibited NSCLC cell proliferation	[105]
	H1975, H1299 + miR-22-3p inhibitor	↓	–	↑	–	–		
	Colorectal cancer cells (SW480, SW620, Caco2, HT29, LOVO, HCT15, HCT116)	↓	–	–	–	–	miR-22 expression was decreased in colorectal cancer cell lines and decreased proliferation and migration of colon cancer cells	[104]
	SW480 + Overexpressed miR-22	↑	–	↓	↓	–		
	SW480 + miR-22 inhibitor	↓	–	↑	↑	–		
	HCC cells (Hep3B, SMMC7721)	↓	–	–	–	–	miR-22 expression was decreased in HCC cell lines and suppressed tumor cell proliferation	[103]
	Hep3B, SMMC7721 + transfected with miR-22	↑	–	↓	–	–		
	Triple negative breast cancer cells (MDA-MB-231, MDA-MB-436, BT-20)	↓	–	–	–	–	miR-22-3p expression was decreased in triple negative breast cancer cells and suppressed cell proliferation and migration	[106]
	MDA-MB-231, MDA-MB-436 + transfected with miR-22-3p	↑	–	↓	↓	–		
	Highly metastatic breast cancer cells (MDA-MB-231, Hs578T)	↑ (vs low metastatic MCF7, T47D)	–	–	–	–	miR-22 expression was higher in highly metastatic breast cancer cell lines and increased cell migration and invasion	[107]
	MDA-MB-231 + miR-22 inhibitor	↓	–	–	↓	–		
	MCF7 + overexpressed miR-22	↑	–	–	↑	–		
	Prostate cancer cells (Ca-HpV-10, DU145, PC3, VCap)	↑	–	–	–	–	miR-22 expression was increased in prostate cancer cell lines	[108]
	*In vivo studies*							
	Mice + Overexpressed miR-22 in NSCLC cells (A549)	↑	–	↓	–	–	miR-22 suppressed lung cancer growth in mice	[102]
	Xenograft colorectal cancer mice + overexpressed miR-22 colorectal cancer cells (SW480)	↑	–	↓	–	–	Overexpression of miR-22 inhibited colorectal cancer growth	[104]
	Mice + overexpressed miR-22 in HCC cells (Hep3B/SMMC7721)	↑	–	↓	–	–	miR-22 suppressed HCC growth in mice	[103]
	Orthotopic immunodeficient mice + overexpressed miR-22 in breast cancer cells (MCF-7)	↑	–	–	↑	–	miR-22 enhanced breast cancer cell metastasis	[112]
	MMTV-miR-22 transgenic mice	↑	–	↑	↑	–	miR-22 promoted breast cancer growth and distant metastasis	
	Orthotopic breast cancer mice (MDA-MB-231, MDA-MB-436) + miR-22-3p	↑	–	↓	–	–	miR-22-3p suppressed breast cancer proliferation	[106]
	Mice + overexpressed miR-22 in prostate cancer cells (DU145)	↑	–	↑	–	–	miR-22 promoted prostate cancer growth	[108]
	*Clinical studies*							
	NSCLC patients	↓	–	–	–	–	Tumor miR-22-3p expression was decreased in NSCLC patients	[105]
	Lung cancer patients	↓	–	–	–	–	Tumor miR-22-3p expression was decreased in lung cancer patients	[102]
	Advanced NSCLC patients	–	↑	–	–	–	Plasma miR-22 level was increased in advanced NSCLC patients	[111]
	Colorectal cancer patients	↓	–	–	↓	↑	Tumor miR-22 expression was decreased in colon cancer and low miR-22 expression was associated with poor survival and liver metastasis	[104, 109]
	Colorectal cancer patients	–	↓	–	–	–	Plasma miR-22-3p level was decreased in colorectal cancer patients	[113]
	Colon cancer patients	↑	–	–	–	–	Tumor miR-22-3p expression was increased in colon cancer patients	[110]
	HCC patients	↓	–	–	–	↑	Tumor miR-22 expression was decreased in HCC and low miR-22 expression was associated with poor survival	[103]
	Triple negative breast cancer patients	↓	–	–	–	–	Tumor miR-22-3p expression was decreased in triple negative breast cancer patients	[106]
	Breast cancer patients	↑ (vs low level)	–	–	–	↓	Elevated tumor miR-22 expression was associated with poor survival in breast cancer patients	[107]
	Prostate cancer patients	↑	–	–	–	–	Tumor miR-22 expression was increased in prostate cancer patients	[108]
	Pancreatic cancer patients	–	↑	–	–	–	Plasma miR-22-3p level was increased in pancreatic cancer patients	[114]

*DLBCL* diffuse large B cell lymphoma, *GBM* glioblastoma, *HCC* hepatocellular carcinoma, *LV* left ventricular, *miR* microRNA, *NSCLC* non-small cell lung cancer, *PE* phenylephrine, *SCID* severe combined immunodeficient

### SerpinA3

SerpinA3, also known as anti-chymotrypsin, is a member of the Serpin superfamily that functions as a serine proteinase inhibitor and plays a role in the function and homeostasis of various organs throughout the body including regulation of blood pressure, insulin sensitivity and inflammatory response [26].

#### SerpinA3 in pathological hearts

An in vitro study using an atherosclerosis model demonstrated an increase in SerpinA3 expression in aortic smooth muscle cells exposed to oxidized LDL [27]. In MI and LV hypertrophy in ATF3-transgenic mice models with tumors, an enhancement in tumor growth and an increase in cardiac expression of SerpinA3 were observed [16, 24]. However, in mice with low-dose PE-induced LV hypertrophy without LV systolic dysfunction, no change in cardiac SerpinA3 expression was found [25]. In clinical studies, increased expression of SerpinA3 has been reported in various cardiac conditions and was associated with poor clinical outcome. Levels of SerpinA3 in the plasma were increased in coronary artery disease (CAD) patients and correlated with the extension of atherosclerosis [27]. In MI patients, plasma levels of SerpinA3 were increased and predicted major adverse cardiac events (MACE) [28]. In HF with reduced ejection fraction (HFrEF), levels of plasma SerpinA3 were also found to increase [29, 30]. In dilated cardiomyopathy (DCM) patients, cardiac expression and plasma levels of SerpinA3 were increased and correlated with poor outcomes [29]. The increased levels of SerpinA3 in DCM patients with LV assist devices (LVADs) were reduced after offloading LVADs [31]. Furthermore, in cases of calcific aortic stenosis, both cardiac and plasma levels of SerpinA3 were also increased [32].

#### SerpinA3 in cancer

SerpinA3 has been implicated in cancer proliferation and invasiveness. SerpinA3 expression was increased in various cancer cell lines including colon cancer and lung adenocarcinoma cell lines [33, 34]. The enhanced expression of SerpinA3 has been shown to promote tumor cell invasion and migration in colon cancer, breast cancer, and glioblastoma cell lines [34–36]. SerpinA3 also promoted the proliferation of colon cancer cells after in vitro exposure [16]. SerpinA3 was also associated with tumor invasiveness as a consequence of remodeling the extracellular matrix, as shown in studies using melanoma and glioblastoma [36, 37]. In mice injected with downregulated SerpinA3 colon cancer cells, a decrease in liver metastasis was demonstrated [34]. In clinical studies, SerpinA3 expression was increased in various tumor tissues, including colon, lung adenocarcinoma, breast and glioma [33–36]. Increased SerpinA3 expression in colon cancer tissues was associated with higher metastasis [34]. Increased expression of SerpinA3 by tumors was also associated with larger tumor size and poor survival in lung adenocarcinoma patients [33]. In addition, high SerpinA3 expression in glioma tissues was shown to be associated with poor survival [36]. Plasma levels of SerpinA3 were elevated in lung cancer patients and were also associated with metastasis [38].

#### SerpinA3 as a link between CVD and cancer

In summary, it has been reported that SerpinA3 is upregulated in association with various types of CVD and enhanced tumor proliferation and invasiveness in several types of cancer. In an MI mouse model which showed enhanced tumor growth, the SerpinA3 expression was increased in cardiac tissue, and an in vitro study demonstrated its role in increasing the growth of colon cancer cells [16]. An ATF3-transgenic mouse model that had enhanced tumor growth was also shown to have increased levels of expression of SerpinA3 in cardiac tissues but not in tumor cells [24]. This evidence suggests that SerpinA3 from CVD could be a cardiokine responsible for the enhanced tumor growth. These reports on SerpinA3 are comprehensively summarized in Tables 4 and 5.

### SerpinA1

SerpinA1, also known as antitrypsin, is also a member of the Serpin superfamily [26].

#### SerpinA1 in pathological hearts

Unlike SerpinA3, the evidence surrounding the expression of SerpinA1 in CVD models is still limited. In a mouse model of MI, cardiac SepinA1 expression was found to be increased [16]. In clinical studies, plasma levels of SerpinA1 were also elevated in MI and HFrEF patients [39, 40]. More studies are needed to validate these findings regarding the level of SerpinA1 in association with CVD.

#### SerpinA1 in cancer

SerpinA1 has been reported to promote and be associated with outcomes in various types of cancer. It has been shown to promote cell proliferation in colon cancer and non-small cell lung cancer (NSCLC) [16, 41]. Additionally, upregulation of SerpinA1 has also been found to promote tumor migration and invasion in various cancer cell lines, including gastric, colon, breast, ovarian and lung cancer cells [41–45]. In mice, upregulated SerpinA1 was shown to promote metastasis of lung adenocarcinoma [43]. In clinical studies, increased expression of SerpinA1 in tumor tissues was associated with larger tumor size, metastasis and poor survival in colorectal and gastric cancer patients [42, 44]. In lung cancer patients, increased SerpinA1 expression was associated with metastasis and poor survival [41, 43, 45]. Additionally, SerpinA1 has been shown to promote lung cancer metastasis through regulation of expression of FN [43, 45].

#### SerpinA1 as a link between CVD and cancer

As mentioned earlier, it has been demonstrated that SerpinA1 promotes growth in various types of tumor, however, the evidence in CVD models is still very limited. Only one report has demonstrated the potential effect of CVD through SerpinA1 on the enhancement of tumor growth. In an MI-induced HF mouse model in which enhanced tumor growth was found, increased cardiac SerpinA1 expression was demonstrated [16]. However, there was no change in cardiac SerpinA1 expression following the heterotopic heart transplant of an MI model, which also reported enhanced tumor growth [16]. Further studies are needed to warrant the potential role of SerpinA1 in this setting. These reports are comprehensively summarized in Tables 4 and 5.

### Periostin

Periostin is a secreted protein that serves as a component of the extracellular matrix and plays a crucial role in cell–matrix interactions [46]. It is associated with transforming growth factor-β (TGF-β) and regulates fibroblast function, contributing to collagen fibrillogenesis, which is involved in cardiac remodeling [46].

#### Periostin in pathological hearts

An in vitro study demonstrated that angiotensin II (Ang II) promoted the expression of periostin in adult rat cardiac fibroblasts [47]. Mice treated with an Ang II infusion developed LV hypertrophy and fibrosis, and an increase in expression of periostin by cardiac tissue was reported [47]. In high salt-induced HT rats, increased cardiac expression of periostin along with cardiac fibrosis was shown [48]. Cardiac expression of periostin was also increased in both MI and hypertensive-induced HF models [49–52]. Studies demonstrated enhanced tumor growth in cardiac remodeling models including TAC-operated mice, low-dose PE-induced HT mice, and ATF3-transgenic mice also reported increased periostin expression in cardiac tissues [23–25]. In clinical studies, increased cardiac expression of periostin was observed in patients with MI and HFrEF [49, 52, 53]. Additionally, levels of periostin were found to be elevated in the plasma in ST-elevation MI (STEMI) patients and were associated with increased CV events and declining LV systolic function [54].

#### Periostin in cancer

As a component of the tumor microenvironment, periostin is one of the matricellular proteins, a group of non-structural matrix components that plays a critical role in tumorigenesis and metastasis [55]. Periostin has been shown to interact with tumor cells and promote cell proliferation, migration, survival, epithelial-mesenchymal transition, and contribute to distant metastasis [55]. An in vitro study showed that incubation with periostin enhanced the proliferation of breast and lung cancer cells [23]. Upregulation of periostin was shown to stimulate lung cancer cell proliferation and migration, promote tumor angiogenesis in breast cancer cells, and promote colon cancer cell survival under stress conditions [56–58]. In mice, upregulation of periostin in breast and colon cancer cells promoted tumor growth and metastasis, respectively [57, 58]. In cancer patients, periostin expression was increased in various types of cancer tissues, including colon, prostate, NSCLC and breast cancer, and was associated with poor survival [57–61]. Increased tumor expression of periostin was also associated with advanced stages in colon, prostate, NSCLC and hepatocellular carcinoma (HCC) patients [56, 58, 59, 62, 63]. Plasma periostin levels were elevated in multiple types of cancer patients, including colon, NSCLC and HCC patients, and were correlated with poor survival [56, 58, 59, 64, 65]. In breast cancer patients, elevated plasma periostin levels were associated with bone metastasis [66].

#### Periostin as a link between CVD and cancer

Evidence demonstrated that expression of periostin in both cardiac tissue and plasma were increased in various CVD models and play a role in tumorigenesis and progression. In a cardiac remodeling mouse model that showed enhanced tumor growth, both cardiac tissues and plasma levels of periostin were reported to be increased, and an in vitro study demonstrated its role in tumor cell proliferation [23–25]. These findings suggest that periostin could be chemokine potentially responsible for enhanced tumor growth in CVD models. The reports on periostin in CVD and cancer are comprehensively summarized in Tables 4 and 5.

### miR-21

#### miR-21 in pathological hearts

The miR-21 was found to be expressed by both cardiomyocytes and cardiac fibroblasts [67]. Previous in vitro studies using neonatal rat cardiomyocytes reported an increased miR-21 expression after exposure to hypertrophic stimuli including PE and Ang II [68, 69]. In various HF mouse models, including β_1_-adrenergic receptor transgenic mice, TAC-induced HF mice, and isoproterenol-induced HF mice, cardiac expression of miR-21 was increased [70]. In cardiac ischemic/reperfusion (I/R) injury mice, miR-21 expression was increased in the infarct region, particularly with regards to cardiac fibroblasts [71]. In an MI rat model, miR-21 expression was upregulated at the border zone but was downregulated in the infarct area during early post-MI, specifically within the first 24 h [72]. Between 3 days to 2 weeks, miR-21 expression, however, was upregulated in both the border and infarct zones, especially in the infarct area in MI mice [73]. In clinical studies, cardiac expression of miR-21 was also increased in end-stage HF patients [70]. Plasma levels of miR-21 were found to be elevated in acute coronary syndrome (ACS), CAD and HFrEF patients [74, 75]. Elevated plasma levels of miR-21 were also associated with a decline in LV ejection fraction and increased NYHA functional status in HFrEF patients [74].

#### miR-21 in cancer

The miR-21 is one of the most closely cancer-related mi-RNAs and is frequently upregulated in a wide range of solid tumors and hematologic malignancies [76, 77]. Several mechanisms have been identified through which miR-21 promoted cancer cell proliferation and migration [76, 77]. Upregulated miR-21 enhanced tumor cell proliferation in various cancer cell lines, including colorectal, NSCLC and gastric cancer [78–80]. Additionally, miR-21 expression enhanced tumor cell migration and invasiveness in colorectal, breast, NSCLC, gastric, glioblastoma and HCC [78–86]. Previous in vivo studies using mouse models showed that miR-21 promoted growth of colorectal and breast cancer [78, 83]. In clinical studies, it has been observed that expression of miR-21 is increased in various cancer tissues including colorectal, breast, lung, gastric, glioma, prostate, HCC and diffuse large B-cell lymphoma [64, 79, 80, 82, 85–88]. High expression of miR-21 by the tumor has been associated with metastasis in colorectal, breast, NSCLC and gastric cancer patients [64, 79, 80, 82]. In colon cancer patients, high tumor miR-21 expression was correlated with poor survival [89]. Elevated plasma miR-21 levels have been observed in breast, NSCLC, prostate, and gastric cancer patients [83, 90–92]. In NSCLC patients, high plasma miR-21 levels have been associated with lymph node metastasis and poor survival [90]. High plasma miR-21 levels have also been associated with advanced disease in prostate cancer patients [92].

#### miR-21 as a link between CVD and cancer

Overall, there is extensive evidence that there is increased expression of miR-21in CVD and that it is linked to tumorigenesis and cancer progression. Despite this evidence, there have still been no studies verifying the causal effect of miR-21 in tumor enhancement in CVD models. Further studies are required to illustrate the mechanistic link and determine the potential role of miR-21 in reverse cardio-oncology. These reports on miR-21 in CVD and cancer are comprehensively summarized in Tables 4 and 5.

### miR-22

#### miR-22 in pathological hearts

It has been demonstrated that miR-22 is related to cardiac remodeling and LV hypertrophy [93]. Previous in vitro studies using neonatal rat cardiomyocytes demonstrated an increase in miR-22 expression after exposure to hypertrophy stimuli, including PE and Ang II [69, 94]. An in vivo study in TAC-induced cardiac hypertrophy mice also demonstrated increased cardiac expression of miR-22 [94]. In clinical studies, both cardiac expression and plasma levels of miR-22 were elevated in HFrEF patients [95, 96]. Elevated plasma miR-22 levels were also associated with an increased risk of CV death [96]. The miR-22-3p is the mi-RNA derived from the 3’ arm of miR-22 [22]. An in vivo study using MI-induced HF mice reported increased cardiac expression and plasma levels of miR-22-3p [18]. A study in HF patients, including both HFpEF and HFrEF, showed that an increased plasma miR-22-3p level was associated with a lower risk of CV events [97]. It has also been reported that plasma miR-22-3p was increased in CAD patients [98, 99]. Conversely, an earlier study reported decreased plasma miR-22 levels in CAD patients [100]. These discrepancies in the expression levels could be explained by differences in patient subgroups and specific type of miR-22.

#### miR-22 in cancer

The mechanistic role of miR-22 in cancer is variable and depends on specific cancer types [101]. It has been shown to have a tumor suppressor role by inhibiting tumor proliferation, invasion, and metastasis in various types of cancer [101]. Previous in vitro studies showed decreased expression of miR-22 in various cancer cell lines including NSCLC, HCC and colorectal cancer and overexpression of miR-22 suppressed cancer cell proliferation and migration in these cancer cell lines [102–104]. In NSCLC, overexpression of miR-22-3p also inhibited cell proliferation [105]. Similarly, in triple negative breast cancer cells, decreased expression of miR-22-3p was observed, and that the overexpression of miR-22-3p could suppress cancer cell proliferation and migration [106]. Previous in vivo studies have also demonstrated the tumor suppressing effect of miR-22 and miR-22-3p in lung, colorectal, HCC and breast cancer mice models [102–104, 106]. Conversely, miR-22 has been reported to promote tumor progression and metastasis in some cancers [101]. Higher miR-22 expression was observed in highly metastatic breast cancer cell lines, and also enhanced cell migration and invasion [107]. Similarly, prostate cancer cells also had increased miR-22 expression [108]. In an orthotopic breast cancer mouse model with miR-22 overexpression and an miR-22 transgenic mice model enhanced breast cancer metastasis was reported [107]. Overexpression of miR-22 also enhanced prostate cancer growth in a mouse model [108].

In clinical studies, decreased tumor expression of miR-22-3p has been reported in various cancers including lung, HCC, and breast cancer [102, 103, 106]. Low miR-22-3p expression was associated with poor survival in HCC patients [103]. Tumor miR-22 expression also decreased in colorectal cancer patients, and low tumor miR-22 expression was associated with liver metastasis and poor survival [104, 109]. However, another study in colon cancer patients reported increased expression of miR-22-3p by the tumor [110]. Conversely, elevation of tumor miR-22 has been reported in prostate cancer [108]. In breast cancer, elevated tumor miR-22 expression was associated with poor survival [107]. In advanced NSCLC patients, plasma miR-22 has been reported to be increased [111].

#### miR-22 as a link between CVD and cancer

A recent study demonstrated that secreted miR-22-3p from pathologic hearts of MI-induced HF mice mitigate the sensitivity of lung cancer to ferroptosis which may be responsible for tumor growth and possible cancer therapy resistance [18]. However, investigation into miR-22 is still very limited. Further studies are needed to better understand the role of decreased ferroptosis sensitivity of cancer induced by miR-22-3p in the pathological heart model. These reports on miR-22 in CVD and cancer are comprehensive summarized in Table 4 and 5, respectively.

## Future perspective and conclusion

**Fig. 3:**
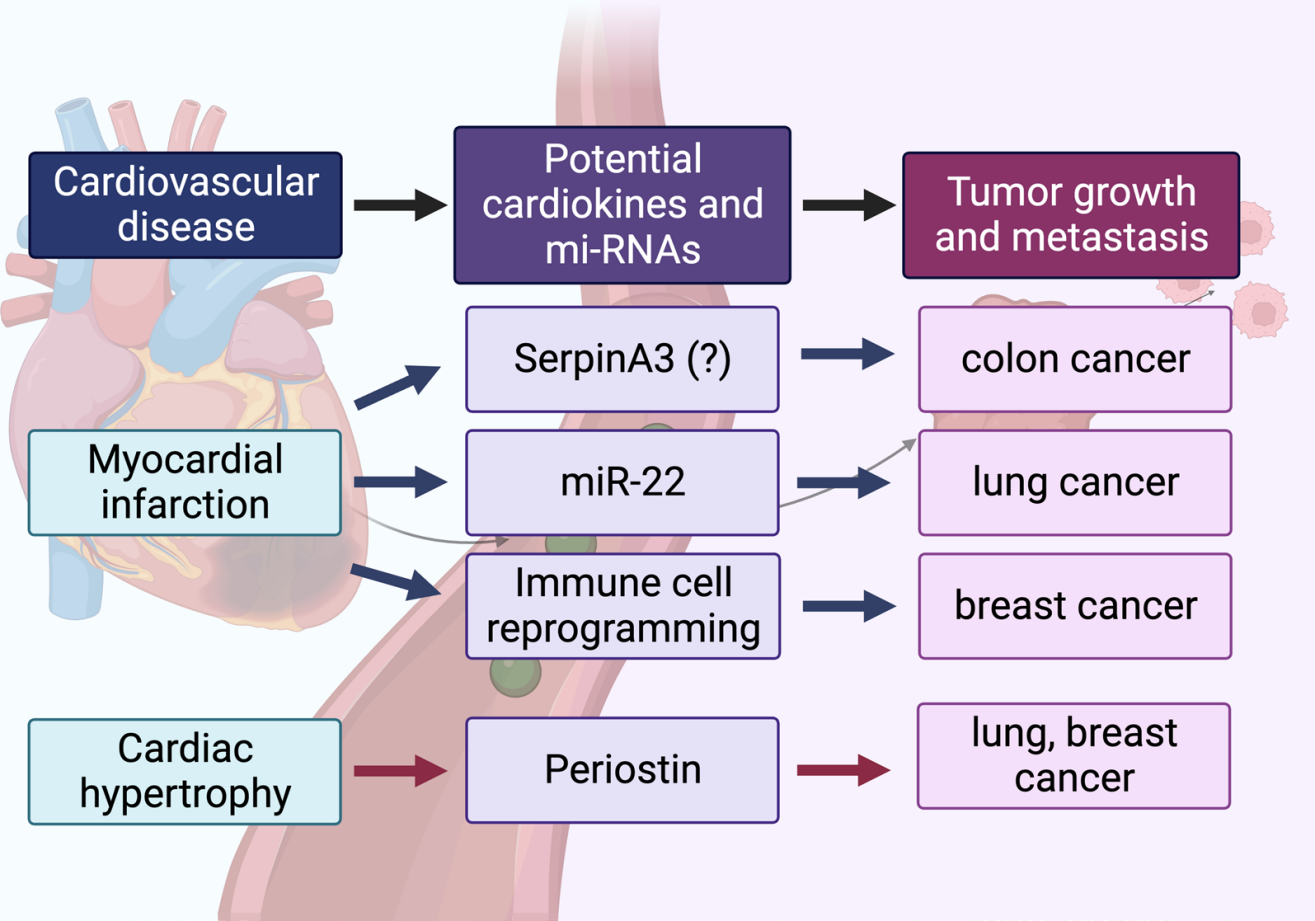
A schematic diagram summarizes the potential direct effects of cardiovascular disease on tumor growth and metastasis. An MI model was shown to promote colon, lung, breast cancer growth and metastasis of breast cancer, with potential cardiokines including SerpinA3, mi-RNAs, and immune cell reprogramming. Cardiac hypertrophy was demonstrated to enhance the growth and metastasis of lung and breast cancer through potential cardiokines, including periostin. Figure created with BioRender.com

There is an increasing body of evidence from both in vitro and in vivo studies to demonstrate possible mechanisms by which CVD directly promotes cancer growth and metastasis. Currently, the effect of CVD on the promotion of tumor growth and proliferation are cancer-type specific and may be mediated via the secretion of several cardiokines, and mi-RNAs and immune cell reprogramming. An MI mouse model showed increased tumor growth and metastasis of colon, lung and breast cancer via possible cardiokines including SerpinA3, and mi-RNAs, and also as a consequence of immune cell reprogramming into an immunosuppressive tumor microenvironment. The mi-RNAs including miR-22-3p in an MI-induced HF model also attenuated the tumor sensitivity to ferroptosis in a lung cancer mouse model. Cardiac hypertrophy also enhanced breast and lung cancer growth and metastasis, which could be mediated by several cardiokines including periostin. The schematic diagram summarizing the current evidence is shown in Fig. 3. Nevertheless, there are still a limited number of studies dedicated to investigating and verifying a causal relationship between CVD and tumor progression. Furthermore, while many cardiokines and mi-RNAs have been shown to be involved in both CVD and cancer, studies that examine their causal relationship remain limited. Further studies for potential cardiokines and mi-RNAs secreted from pathological heart tissues are required. Moreover, the possible mechanisms involved in the systemic disturbance from CVD and the secreting factors from other organs as a result of hemodynamic changes or neurohormonal responses could also play a role in cancer exacerbation and require further investigations. A better understanding of the pathophysiology of reverse cardio-oncology could contribute to future risk stratification and therapeutic prevention for subsequent cancer progression in CVD patients.

## Data Availability

Not applicable.
